# Developing a Prolamin-Based Gel for Food Packaging: In-Vitro Assessment of Cytocompatibility

**DOI:** 10.3390/gels9090740

**Published:** 2023-09-12

**Authors:** Franziska Trodtfeld, Tina Tölke, Cornelia Wiegand

**Affiliations:** 1 Department of Dermatology, Jena University Hospital, Friedrich Schiller University Jena, Am Klinikum 1, D-07747 Jena, Germany; 2 INNOVENT e.V., Prüssingstraße 27 B, D-07745 Jena, Germany; tt@innovent-jena.de

**Keywords:** food packaging, composite gels, biodegradable polymers, cytocompatibility, in vitro testing

## Abstract

Growing environmental concerns drive efforts to reduce packaging waste by adopting biodegradable polymers, coatings, and films. However, biodegradable materials used in packaging face challenges related to barrier properties, mechanical strength, and processing compatibility. A composite gel was developed using biodegradable compounds (prolamin, d-mannose, citric acid), as a coating to increase the oxygen barrier of food packaging materials. To improve gel stability and mechanical properties, the gels were physically cross-linked with particles synthesized from tetraethyl orthosilicate and tetramethyl orthosilicate precursors. Additionally, biocompatibility assessments were performed on human keratinocytes and fibroblasts, demonstrating the safety of the gels for consumer contact. The gel properties were characterized, including molecular structure, morphology, and topography. Biocompatibility of the gels was assessed using bioluminescent ATP assay to detect cell viability, lactate dehydrogenase assay to determine cell cytotoxicity, and a leukocyte stimulation test to detect inflammatory potential. A composite gel with strong oxygen barrier properties in low-humidity environments was prepared. Increasing the silane precursor to 50 wt% during gel preparation slowed degradation in water. The addition of citric acid decreased gel solubility. However, higher precursor amounts increased surface roughness, making the gel more brittle yet mechanically resistant. The increase of precursor in the gel also increased gel viscosity. Importantly, the gels showed no cytotoxicity on human keratinocytes or fibroblasts and had no inflammatory effects on leukocytes. This composite gel holds promise for oxygen barrier food packaging and is safe for consumer contact. Further research should focus on optimizing the stability of the oxygen barrier in humid environments and investigate the potential sensitizing effects of biodegradable materials on consumers.

## 1. Introduction

The food packaging industry is continuously looking for ways to improve packaging materials to ensure optimal durability and strength for food products’ shelf lives. The food packaging industry’s primary concerns are to ensure the safety of food contents and to prolong their shelf life by protecting them from spoilage, oxidation, and external contaminants. But in response to growing environmental concerns, there is a concerted effort to additionally reduce packaging waste through the increased use of biodegradable polymers, coatings and films [1,2]. Degradable materials used in packaging face challenges in terms of their barrier properties, mechanical strength, and processing compatibility [3,4]. Key considerations in the development and introduction of these materials are: (i) ensuring adequate barrier properties to protect the packaged products from oxygen and the following degrading oxidation reactions; (ii) achieving sufficient mechanical strength for durability; and (iii) finding processing methods that are compatible and efficient. Nanocomposite films and coatings containing inorganic particles have been investigated for their potential applications in food packaging [5,6,7,8]. These nanocomposites have exhibited satisfactory barrier properties, attributed to the particles effectively impeding the diffusion of molecules [9,10,11]. In addition to their exceptional barrier performance, the incorporation of nanomaterials can enhance stability, mechanical strength, and overall durability in degradable food packaging coatings [12,13]. Additionally, the incorporation of nanomaterials serves to enhance the water resistance of the inherently hydrophilic degradable polymers. This aspect holds particular significance, considering that the majority of food products characteristically contain higher moisture levels [14,15]. A composite gel with an oxygen barrier for food packaging was developed using in-situ sol-gel polymerization in a matrix of biodegradable polypeptides, polysaccharides, and organic acids. Controlled sol-gel conditions (time, temperature, pH) created a semi-solid gel with a continuous network of nanoparticles and pores [16,17]. Polar groups in proteins enhanced the oxygen barrier by slowing down oxygen diffusion [2,18]. D-mannose served as a carrier, and citric acid as a catalyst for the silane precursor reaction [19,20]. Physical cross-linking with particles synthesized from tetraethyl orthosilicate and tetramethyl orthosilicate improved gel stability [21,22]. Comprehensive analysis included molecular structure, morphology, and topography investigations.

At the same time, new materials for food packaging potentially coming into contact with the skin need to be assessed for biocompatibility and eliciting inflammatory reactions and skin irritation in humans [23]. Natural polymers, derived from sources such as plants or animals, may contain sensitizing or irritating components that can initiate inflammatory reactions in susceptible individuals [24,25,26]. These reactions can range from mild symptoms, such as skin irritation, to more severe consequences, including tissue damage and health complications [27,28,29]. Identifying and evaluating skin irritants in natural polymers and their derivatives is necessary for consumer safety. To develop safe materials, the synthesized gels underwent a series of biological assays. Cytotoxicity was assessed using in vitro assays with human keratinocytes and fibroblasts exposed to various extraction mediums that mimic environmental conditions. Additionally, an initial evaluation of inflammatory potential was conducted by detecting sulfidoleukotrienes following leukocyte stimulation [30]. This preliminary assessment allows to gain insights into the gels initial response to immune cells and potential initiates of inflammation [31].

This study examines the physical and chemical properties of a novel prolamine silica composite gel designed as a coating to enhance oxygen barrier properties in food packaging materials. Additionally, it proposes a testing method to evaluate cytocompatibility for biodegradable materials, thus ensuring the safety by utilization.

## 2. Results and Discussion

Sol-gel coatings (ProMa) with different amounts precursor consisting of tetramethoxysilane (TMOS) and tetraethoxysilane (TEOS) were obtained by in situ polymerization in a 70% ethanol solution containing extracted wheat gluten prolamins, d-mannose and citric acid.

### 2.1. IR Spectroscopy Studies

Prolamins extracted in 70% EtOH/H_2_O *v*/*v* % from gluten, the storage protein unit of wheat and the coating with the polymer matrix and 50 wt% precursor were analyzed with FT-IR spectroscopy to investigate molecular structure of the gel.

The proposed network structure of the material consists of different types of chemical bonding, including peptide bonds within the polypeptides, glycoside bonds of d-mannose, and intra- and intermolecular hydrogen bonds. Additionally, ionic bonds between citric acid and amide groups, as well as potential hydrogen bonding, contribute to cross-linking. It is also hypothesized that the silica particles may possess a negatively charged surface, allowing for the adsorption of polymers due to favorable surface area and energy ratios [32,33]. These various bonding interactions result in a network structure, providing mechanical stability and reinforcing the material’s overall properties. However, further research is needed to confirm and fully understand the significance of these bonds in the network.

**Figure 1:**
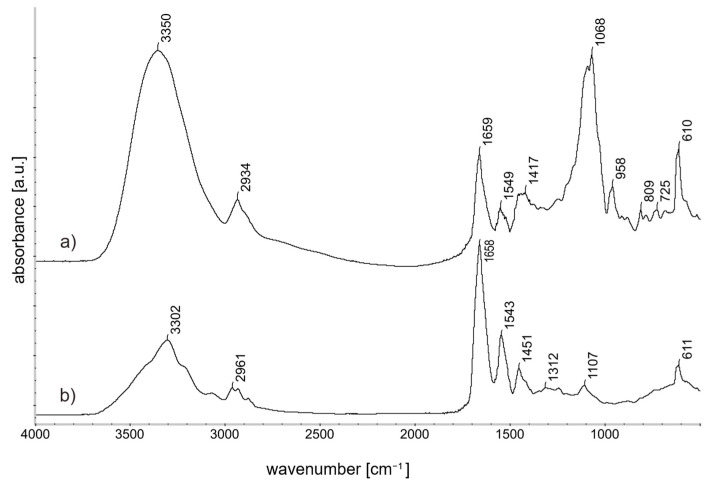
FT-IR spectra (4000–500 cm^−1^ spectrum): (**a**) ProMa 50 wt% precursor gel on silicon wafer (**b**) Prolamins extracted from Gluten Powder (>75%) on silicon wafer.

In Figure 1 the presence of the amide bonds I and II in the polypeptides within the polypeptide structure of the coating is clear from the distinct peaks observed at 1650 cm^−1^ and 1550 cm^−1^, respectively [34].

The peak at ~950 cm^−1^ and the peak at 1000–1200 cm^−1^ (C-O-C stretching) can be attributed to the glycosidic linkage in a polysaccharide. The peaks 809 cm^−1^ and 1068 cm^−1^ are characteristic IR signals of the d-mannose [35]. Silica bonds are superimposed by the matrix bonds in the spectrum, but can be determined at 1096 cm^−1^ and 960 cm^−1^ when the polymer matrix is subtracted from the spectra [36]. The peak around ~3300 cm^−1^ is characteristic for –OH groups and the ~2900 cm^−1^ for saturated hydrocarbons [37,38].

### 2.2. Tensile Strength Examinations of Composite Gels

**Figure 2:**
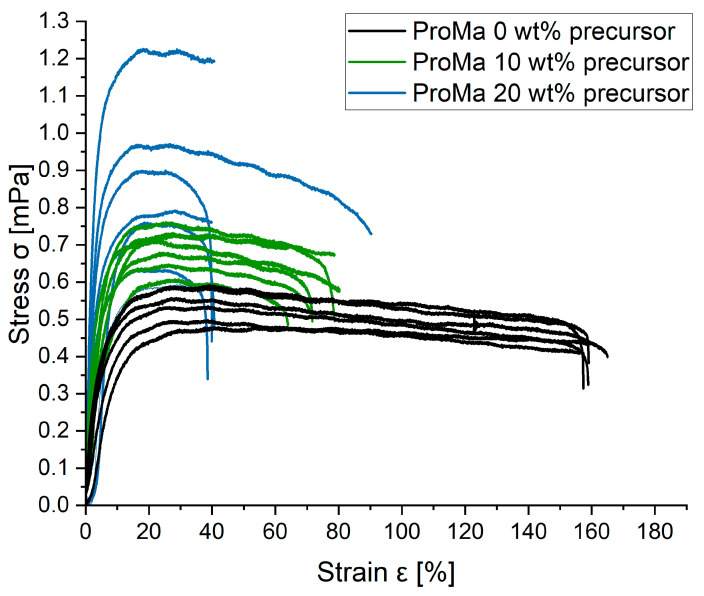
Stress σ [mPa] as a function of Strain ε [%] for ProMa with 0/10/20 wt% precursor.

Texture analysis was performed to evaluate additional material properties of structure and composition, which are relevant for processability, coating stability and material strength. The strain-stress curve (Figure 2) of a material can show the mechanical properties such as elasticity and strength. In this study, the gels were cast dried and analyzed as thin foils to determine differences between the coating compositions. The results are not directly transferable to conclude the material behavior if the gels are coated onto different materials but allow a basic comparison between the gel compositions tested.

The strain stress curve shows that the polymer matrix ProMa 0 wt% precursor is an elastic material that exhibits a long necking phase before the material breaks [39]. As the amount of silica precursor for this material is increased in ProMa 10 wt% precursor and ProMa 20 wt%, the elasticity decreases while the yield strength of the material increases. From this, it can be concluded that the material becomes harder and more resistant to mechanical forces [40]. On the other hand, the fracture point is reached at a lower load. This means that the material becomes more brittle and less ductile [41,42].

The Young modulus was fitted to the strain stress curves and the average of six curves was taken (Table 1). The Young modulus is a material parameter that describes the proportional relationship between stress and strain during the deformation of a solid body with linear-elastic behavior and serves as an indicator of its stiffness and strength.

A higher Young modulus as seen in ProMa 20 wt% precursor compared to ProMa 0 wt% precursor, signifies a greater ability to resist deformation under external pressure, indicating increased stiffness and strength. It reflects the sample capacity to withstand stress without undergoing permanent deformation. This correlation supports that the incorporation of silica precursor contributes to the reinforcement and improved mechanical resistance of the polymer matrix [17,18].

This data emphasizes the observed trend that as the silane precursor concentration within the gel rises, intermolecular chain entanglement becomes more pronounced. The rigidity of the gel increases and the movement of molecular chains of the degradable matrix decreases [43]. This heightened entanglement is manifested in reinforced gel interactions, as evidenced by an increase in tensile strength and a corresponding decrease in elongation at break [14,44]. Hence, it becomes imperative to determine the ideal precursor quantity that can enhance the mechanical properties, specifically elevating tensile strength while keeping material brittleness within acceptable limits.

### 2.3. Atomic Force Microscopy Analysis

**Figure 3:**
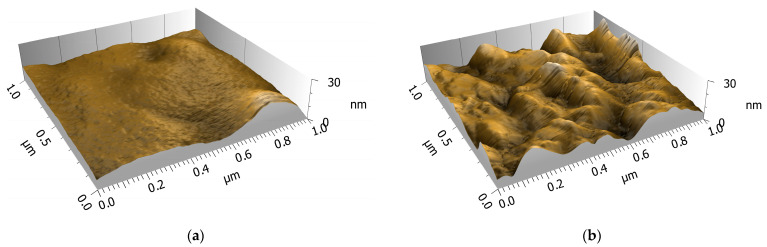
AFM picture of (**a**) ProMa 0 wt% precursor (**b**) Proma 50 wt% precursor.

The analysis of the surface topography and structure of the gels was conducted by atomic force microscopy (AFM). Silica particles have a rough, irregular surface structure, and when dispersed in a polymer matrix [45], they produce a roughened surface (Figure 3).

The arithmetic mean of the surface level (Sa) increased from 3.18 nm for ProMa with 0 wt% of precursor to 9.48 nm for ProMa 50 wt% precursor (measured over a 10 µm × 10 µm area) [46]. Increased particle aggregation, which is present in samples with high silane precursor can lead to an increase in surface roughness, which result in the increased material brittleness observed in texture analysis (Figure 2) [47,48]. This supports the restricting of molecular chain movement and increased entanglement. The roughness increases the surface area of the material, which promotes the adhesion of additional coating. At the same time, the material becomes more hydrophilic, meaning it attracts more water. This could be beneficial for distribution and adhesion of additional coating, such as a water vapor barrier [49].

### 2.4. Scanning Electronic Microscopy Analysis

**Figure 4:**
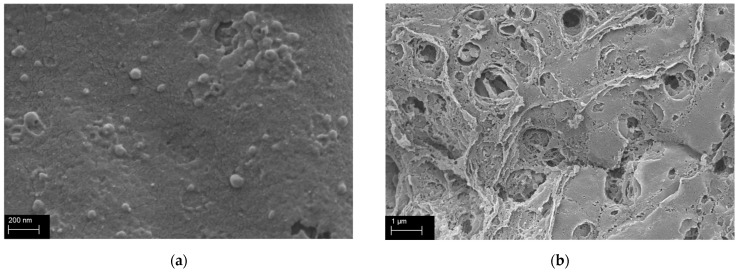
SEM images of ProMa + 20 wt% precursor (**a**) surface and (**b**) fracture edge, dried with CO_2_ critical control point method and gold sputtered.

The material surface and morphology of cross sections were determined using scanning electronic microscopy (SEM). The distribution of particles within the sample appears to be homogeneous through the polymer matrix, indicating an even dispersion throughout the material. Figure 4 provides insight into the size estimation of the silica particle agglomerates, which is estimated to be around 100 nm. This finding suggests that the silica particles have formed relatively uniform agglomerates within the material.

### 2.5. Rheological Investigations of Composite Gels

**Figure 5:**
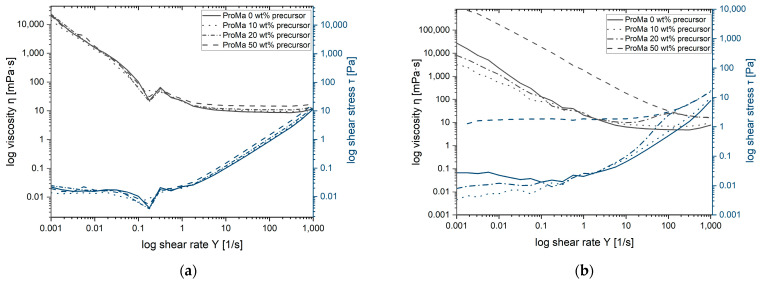
Viscosity curve of ProMa + 0/10/20/50 wt% precursor (**a**) directly after synthesis (**b**) median of measuring 1 d, 3 d and 7 d after synthesis.

The rheological properties were analyzed to obtain data for viscosity, flow behavior and altering of the gel, to understand the processability and applications possibility. By examining the data from viscosity measurements, changes in the material’s flow behavior over time can be observed, reflecting the altering rheological properties [50]. This information is crucial to understanding the material’s process ability and gelling time. The rheological properties and viscosity of the polymer matrix vary depending on the amount of silica precursor present during the modification process [51,52]. The viscosity of all samples decreased with increasing shear rate, which demonstrates the shear thinning effect [53,54]. This phenomenon can be attributed to the entanglement of polymers within the network and their subsequent detanglement in response to applied shear stress. The viscosity for the gel ProMa 0 wt% precursor does decrease from 9.4 mPa∙s after 1 d to 6 mPa∙s at 7 d whereas for the ProMa 50 wt% precursor the viscosity increases from 8.76 mPa∙s after 1 d up to 28.3 mPa∙s at 7 d. Figure 5 provides a visual representation of the viscosity immediately after synthesis and the median viscosity measured over a one-week period after synthesis.

At very low shear rates, the viscosity tends to infinity. After the shear-thinning valley, a slight shear thickening effect is observed at very high shear rates. This shear thickening phenomenon is attributed to the increased frequency of particle collisions within the solution, even in samples without addition of silica precursor, suggesting that it is not solely caused by formed particles.

Furthermore, the addition of up to 50 wt% silica precursors to the solution results in a slightly increased viscosity trend, in general, the materials behave in the same ways as a thin liquid and are very easy to process. During one week of gel altering, the viscosity curve intercepts at a log shear rate of 100. If the flow rate and viscosity overlap, it means that at low shear stress (low flow rate) the viscosity of the material is high indicating that it is thick and viscous. However, with increasing shear stress (higher flow rate), the viscosity decreases, and the material behaves more like a thin fluid [53]. The decline of polymer entanglement in ProMa with 50 wt% precursor indicates a shift towards silica network formation, which will increase the amount of chemical bonding in the gel. This leads to increased viscosity, which is crucial for coating processes [54]. Additionally, silica particles aggregate, strengthening the gel and further elevating viscosity [33]. This viscosity change highlights distinct processing times, depending on precursor concentration. Lower concentrations of precursor such as ProMa 10 wt% and ProMa 20 wt% are not showing a strong viscosity increase, this could be due to reduced aggregation and therefore less crowding, improved electrostatic repulsions and increases solvent interactions [55,56]. This results in longer shelf life compared to ProMa 50 wt% precursor, underscoring viscosity’s role in solution stability.

### 2.6. Water Stability

**Figure 6:**
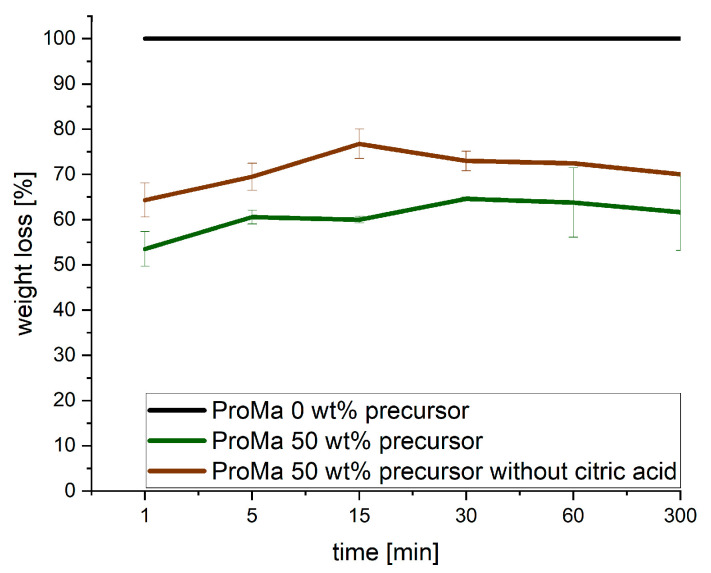
Weight loss [%] over time [min] of ProMa 0/50 wt% precursor and ProMa 50 wt% precursor without adding citric acid during synthesis. A cross-linking of the components chemically and not just physically could increase mechanical stability and decrease solubility of the gel in water. Chemical cross-linking can also make the material less susceptible to natural degradation processes. Microorganisms that aid in the biodegradation of polymers might find it more challenging to break down the material due to the increased cross-linking, leading to reduced biodegradability and change the environmental impact of the material.

The water stability of the gels was determined to analyze if the stability of the degradable gel coating could be improved upon gel alteration by incorporation of silica. The amount of silica precursor also directly affects the coating’s ability to dissolve or disperse in water [57]. Higher concentrations of silica precursor as in inorganic material generally result in reduced water solubility, while lower concentrations may lead to increased solubility and influenced functionality, as it is demonstrated in Figure 6 [58,59]. The decline in solubility of the gels containing 50 wt% precursor can be attributed to a substantial increase in hydrogen bonding. These interactions occur between the SiO_2_ particles and the degradable matrix, leading to a reduction in both the mobility of the polymer chains and the accessibility of hydroxyl groups. This decrease in solubility is a result of these intricate hydrogen bonds forming, which restrict the overall movement of the polymer chains and limit the availability of hydroxyl groups within the system [60]. Integration of silica precursor for 10 wt% and for 20 wt% did not show a decrease in solubility of the gel matrix, concluding that the amount of silica particles formed is not sufficient for available hydroxyl groups of the polymer chains. The incorporation of citric acid into the polymer matrix plays a significant role in enhancing the water stability of the material. Observation shows that with citric acid incorporation decreases the weight loss further from around 60% to around 50% weight loss. Citric acid is used to lower the gel’s pH to 3. In this pH range, silica particles typically reach their point of zero charge (PZC), which typically falls within the pH range of 2 to 4 [61]. This results in a neutrally charged surface that demonstrates a strong affinity for adsorbing anionic polymers, such as prolamins. However, it’s important to note that prolamins, which generally carry a net negative charge, may be less likely to adsorb onto the silica particle surface in a pH 3 environment. This is due to the fact that at pH 3, the carboxyl groups of the amino acids in prolamins are likely to be protonated, resulting in a reduction or neutralization of their negative charge. As a result, the electrostatic attraction between prolamins and the silica surface may be diminished, making adsorption less likely. In conclusion citric acid’s hydrophilic properties introduce more hydroxyl groups, forming hydrogen bonds with neutral silica particle surfaces forming a sturdy three-dimensional network in the matrix that prevents water-induced dissolution [62,63].

### 2.7. Oxygen Permeability of Composite Gels on PET

**Figure 7:**
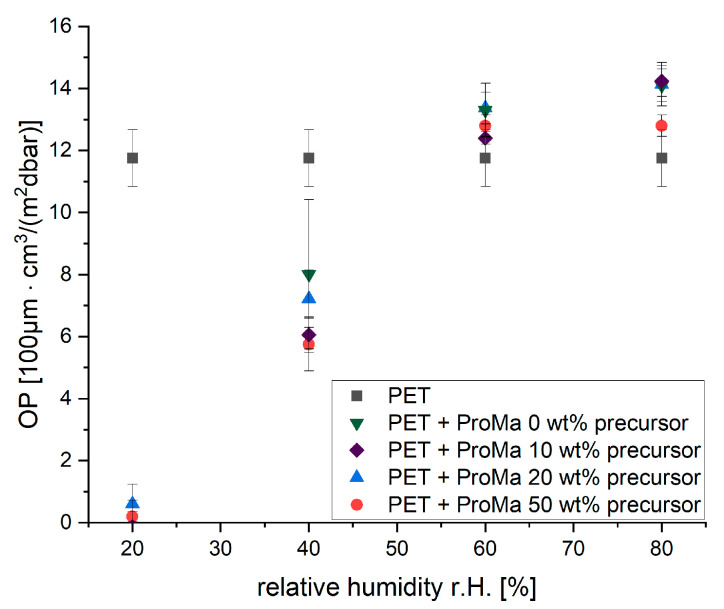
Oxygen Permeability (OP) for PET+ ProMa 0/10/20/50 wt% precursor at different relative humidities 20–80 r.H. [%].

The oxygen permeability was tested of the composite gel coated onto a matrix (PET), to determine if the gels increase the oxygen barrier properties of the matrix material at different relative humidities (r.H.) (Figure 7).

The gels increase the oxygen barrier properties of PET [64,65] in low r.H. environments significantly, but the additional barrier properties cannot be sustained in higher r.H. environments over 60% r.H. When r.H. is increased, there are more water molecules in the surrounding environment. In the case of a polymer matrix, water molecules are attracted to the polar groups within the matrix due to the presence of polar or hydrophilic regions. This attraction leads to the adsorption of water molecules onto the polymer matrix. As water molecules adsorb onto the polymer matrix, they can create gaps or voids within the structure. These gaps act as channels through which other molecules, such as oxygen molecules, can enter. Oxygen permeation through the polymer matrix can be problematic in certain applications [1,66]. The higher the relative humidity and the more water molecules adsorbed, the greater the potential for gaps and increased permeation of oxygen through the matrix [67]. The addition of silica to the polymer matrix plays a vital role in improving the water stability of the material. A subtle reduction in oxygen permeability, noticeable at 80% relative humidity, is evident in gels comprising 50 wt% precursor, while no discernible difference is observed in gels with precursor concentrations ranging from 0 wt% to 20 wt%. Silica particles, known for their hydrophilic properties, effectively act as a barrier against water permeability [32] when incorporated into the polymer matrix. Consequently, the incorporation of silica greatly improves the durability and longevity of the polymer matrix in moisture-sensitive conditions. As can be seen in Figure 7, the oxygen permeability decreases only slightly compared to the polymer coating without precursors. The incorporation of silica precursors leads to an improvement in water stability but does not prevent the formation of voids in the matrix, even though silica incorporation has been reported to improve the oxygen barrier significantly [68,69]. In order to increase the stability of the oxygen barrier in humid environments, it would be useful to increase the crystallinity of the material and decrease the amount of polar groups so that the oxygen barrier remains intact but the attraction to water might decrease [70]. Tailoring particle shape during synthesis could greatly enhance oxygen barrier performance. A lamellar morphology proves more effective in improving barrier properties than spherical particle [71,72]. This also suggests a promising approach for enhancing oxygen barrier stability in high-humidity environments [73]. In summary, higher r.H. does lead to the adsorption of water molecules to the polymer matrix, creating gaps within the structure. These gaps allow oxygen molecules to permeate through the matrix, reducing the material’s overall effectiveness as an oxygen barrier. Therefore, multilayer oxygen barriers and water vapor barriers would be the best option for sufficient food packaging with this gel coating [49].

**Cytocompatibility**

### 2.8. In-Vitro Cytotoxicity Testing

**Figure 8:**
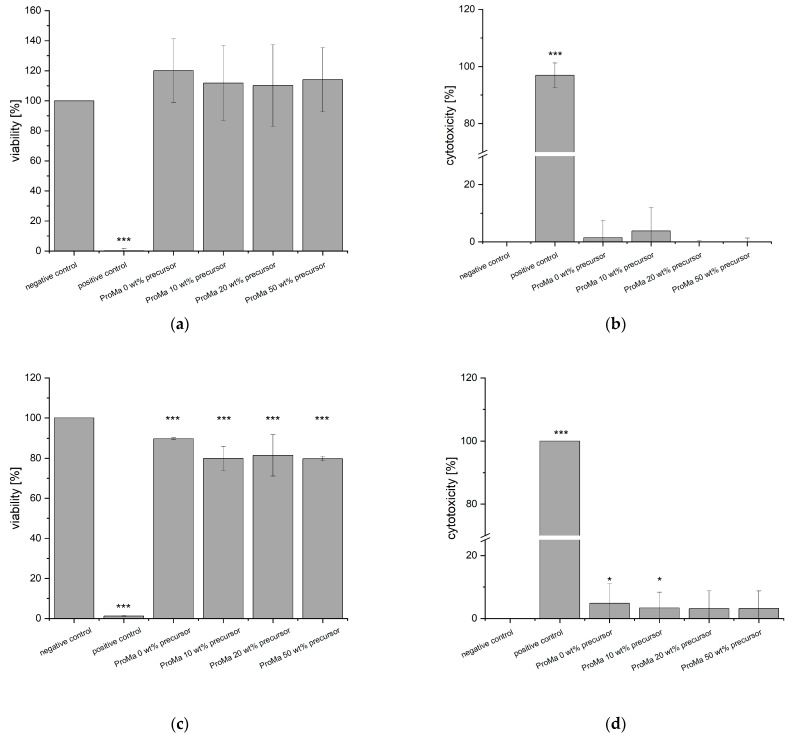
(**a**) cell viability [%] of HaCaT cells exposed to sample extracts in DMEM for 24 h determined with bioluminescent ATP assay (**b**) cytotoxicity [%] of HaCaT cells exposed to sample extracts in DMEM for 24 h, determined with LDH-Assay (**c**) cell viability [%] of Fibroblasts exposed to sample extracts in DMEM for 24 h, determined with bioluminescent ATP assay (**d**) cytotoxicity [%] of Fibroblasts exposed to sample extracts in DMEM for 24 h, determined with LDH-Assay. Asterisks indicate significant deviations from the negative control (* *p* < 0.05; *** *p* < 0.001).

The material extracts were subjected to testing in accordance with standard conditions specified in DIN EN ISO 10993-5 [74], which is primarily intended for medical products and demands an incubation between 24 h and 72 h [75]. However, since food packaging materials typically have shorter contact times compared to medical products, the materials were incubated with cell medium for a 24-h period to allow interaction with the cells. This modified incubation period ensures that the materials have sufficient exposure within a timeframe that more closely matches their contact time in food packaging applications. Subsequently, the extracts were assessed for cellular triphosphate (ATP) content and lactate dehydrogenase release to determine effects on cell viability as well as cell cytotoxicity (Figure 8).

Remarkably, none of the tested materials exhibited any cytotoxic effects, which was confirmed by the lactate dehydrogenase assay. However, it is worth noting that the ATP concentration in fibroblasts decreased slightly, which may be due to reduced fibroblast proliferation associated with the presence of citric acids in the extract. It is known that fibroblasts are sensitive to specific organic acids [76]. However, this sensitivity is concentration and acid dependent. The pH of the cell medium solution after extraction was measured for control and was determined at pH ~8.0 for fibroblasts. Since the citric acid only slightly affected cell proliferation and no direct cytotoxic effect was observed, the results were considered acceptable to continue further investigations.

**Figure 9:**
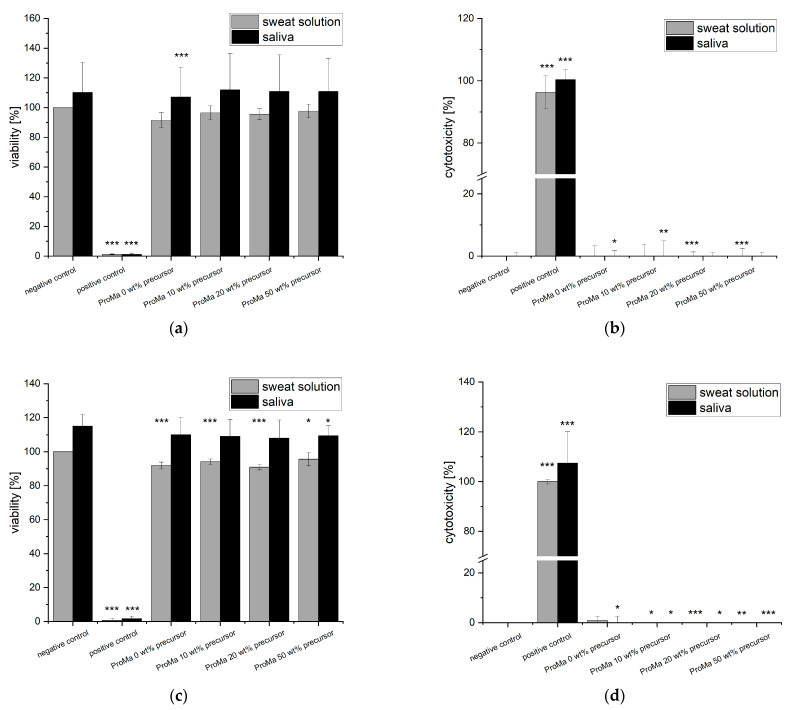
(**a**) cell viability [%] of HaCaT cells exposed to sample extracts in artificial sweat solution (SSL) and artificial saliva (SV) for 24 h, determined with bioluminescent ATP Assay (**b**) cytotoxicity [%] of HaCaT cells exposed to sample extracts in SSL and SV for 24 h, determined with LDH Assay (**c**) cell viability [%] of Fibroblasts exposed to sample extracts in SSL and SV for 24 h, determined with bioluminescent ATP Assay (**d**) cytotoxicity [%] of Fibroblasts exposed to sample extracts in SSL and SV for 24 h, determined with LDH Assay. Asterisks indicate significant deviations from the negative control (* *p* < 0.05; ** *p* < 0.01; *** *p* < 0.001).

Since the material may be exposed to skin cells under various conditions, it was important to investigate the effects of human sweat and saliva on the extraction of specific compounds. To achieve this, extracts of the material were prepared using artificial saliva (SV) and artificial sweat solutions (SSL). The same tests that were conducted with the cell medium were then performed with these extracts (Figure 9). Subjecting the materials to artificial saliva and artificial sweat solutions aimed to simulate more realistic conditions for use and evaluate any potential cytotoxicity. These additional tests provide a comprehensive assessment of the material’s cell compatibility, considering the potential interactions with skin cells in the presence of specific compounds found in human sweat and saliva. The results of these tests will contribute to a deeper understanding of the gels performance and safety in real-life scenarios.

No cytotoxic effects for the material extracts were detected in either the sweat solution or the artificial saliva, which indicates their cell compatibility. However, a slight decrease in fibroblasts was observed when exposed to the sweat solution extracts, which may be due to the presence of citric acids in combination with the lower pH of the sweat solution (pH~5.5) that inhibits cell proliferation [77]. There can be several reasons for this, as the pH affects various cell components (i.e., the enzymatic activity, the DNA stability and replication, the protein function, and the cellular metabolism) and can also cause cell cycle arrest [78].

**Figure 10:**
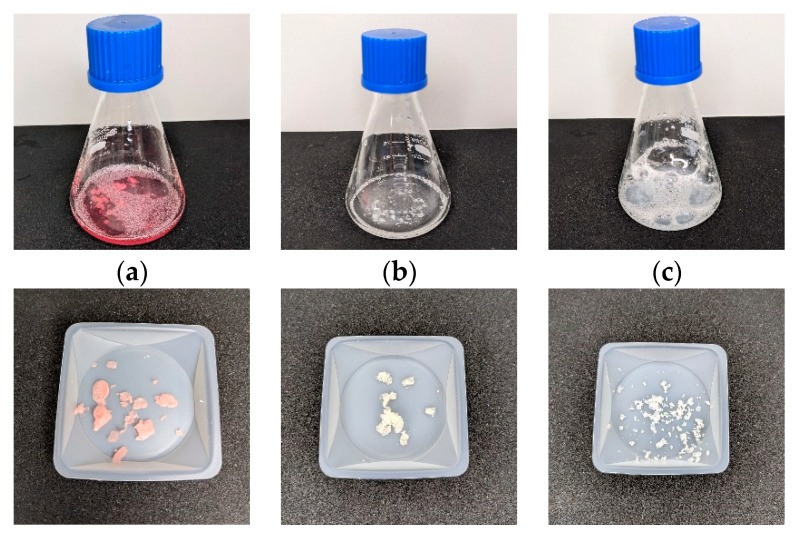
Extract solutions (**a**) DMEM, (**b**) artifical sweat solution, and (**c**) artificial saliva after 24 h, 37 °C and the sample after supernatant removal.

Interestingly, increased cell proliferation was observed in the artificial saliva (pH 6.8). Exposure to saliva can result in improved cell growth and proliferation, due to growth factors, protease inhibitors and a humid environment [79,80]. Since artificial saliva is not a complete replica of human saliva, the constituents are mainly sodium chloride, potassium chloride, potassium thiocyanate, potassium dihydrogen phosphate and urea [81,82,83,84]. These electrolytes play an important role in maintaining the balance of fluids in the body and are involved in various bodily functions. It appears that the saliva acts as a buffer to neutralize organic acids. When acids are present in the mouth, saliva helps maintain the pH balance by neutralizing them [82]. Since the citric acid is the component that lowers the pH and can hinder cell proliferation, buffering this component in addition with a dense nutrient supply for the cells may result in increased cell proliferation. In addition, the artificial saliva incorporates urea, which can disrupt protein structures. This leads to differences in the materials degradation depended on the extraction medium, shown in Figure 10. This supports the hypothesis that the conditions for testing must be adapted to the intended purpose, to enable accurate safety assessment of the material. Protein structure disruption can produce free amino acids and smaller peptides, which can influence cell proliferation. Especially glutamine is a crucial amino acid for cell metabolism and is required for the synthesis of nucleotides, proteins, and other cellular components. It plays a role in supporting the energy needs of rapidly proliferating cells [85,86].

### 2.9. In-Vitro Inflammation Potential Testing

Another important factor for asserting consumer safety of new materials, especially those which are based on natural polymers, is the inflammatory potential [29,87]. Inflammation is a natural defense mechanism, but excessive or prolonged inflammation can lead to tissue damage and other health complications. Testing materials for their potential to induce inflammation helps to ensure that they do not cause harmful effects when exposed to living tissues or cells.

**Figure 11:**
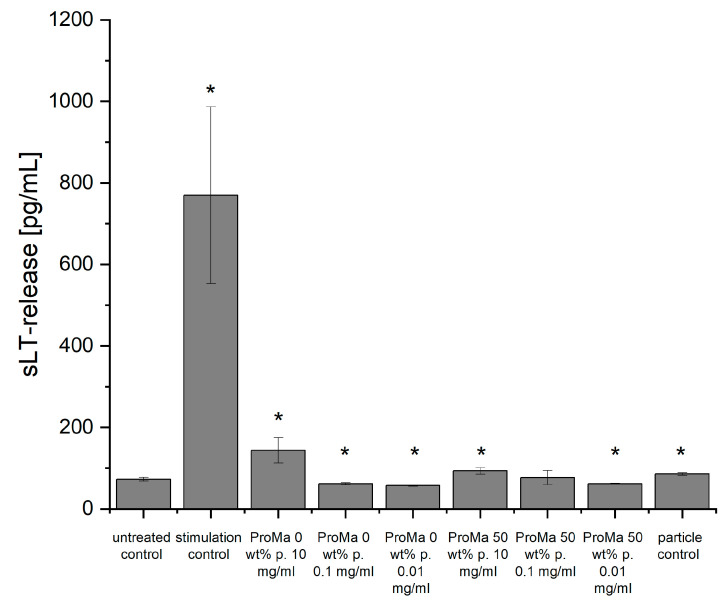
Concentrations of sulfidoleukotrienes (sLT) in (pg/mL). For the leukocyte stimulation assay, the measurements were performed in triplicate (n = 3) for each individual and in duplicate (n = 2) for ELISA. Asterisks indicate significant deviations from the untreated control (* *p* < 0.05).

During inflammation, various cells, particularly leukocytes such as mast cells and eosinophils, produce and release leukotrienes [88]. Upon stimulation by inflammatory signals, the cell membrane phospholipids release arachidonic acid. The arachidonic acids activates the 5-lipoxygenase, which converts arachidonic acid into a reactive intermediate, being then further processed to leukotriene B4 (LTB4) and the cysteinyl leukotrienes (LTC4, LTD4, and LTE4) [89,90]. The produced leukotrienes contribute to the inflammatory response by promoting vasodilation and increasing vascular permeability, as well as attracting and activating immune cells [91]. They act as signaling molecules, homing other leukocytes to the site of inflammation and promoting the release of other inflammatory mediators, such as cytokines [92]. The levels of leukotrienes (LTC4, LTD4, and LTE4) in the affected tissues or body fluids can be measured to assess the extent and severity of inflammation. An immunoassay technique [31] was used to measure the release of sulfidoleukotrienes (sLTs) from isolated leucocytes after incubation with the respective material extracts (Figure 11).

The concentrations of sLTs (expressed in picograms per milliliter, pg/mL) were determined. A reading of 100 pg/mL above the untreated control indicates a significant inflammatory stimulation [93,94]. However, none of the tested materials resulted in a peak concentration that high. The polymer matrix ProMa 0 10 mg/mL led to a slight increase in the sLT production, which could be an effect of the sample’s degradation into the buffer and the cell interaction with polypeptides and organic acids. As the assay components probably stabilize the pH sufficiently, the slight increase in sLT release might be caused by degradation of the sample so that the components would be free to interact with the leukocytes. The samples with incorporated silica demonstrated greater withstanding to dissolution. However, they also release ions into the buffer that reduce the pH. The materials were milled to yield small particles and formed an emulsion with the buffer. Since particles can lead to an increase in sLT production due to physical disruption of the cell membrane, a particle control was also prepared. There is a correlation between the leukocyte stimulation assay and skin sensitization testing [95]. By adopting it to safety evaluations, an effective screening procedure for testing of the inflammatory potential of materials can be established, thereby promoting consumer safety. The comprehensive evaluation of materials for their inflammatory potential and skin sensitization is crucial in ensuring the safety and efficacy of materials used in the food industry. This testing approach helps identify potential risks, allowing for the development of safer and more biocompatible materials and products. As a result, it plays a pivotal role in enhancing consumer trust and ensuring the overall quality of food-related materials and applications.

## 3. Conclusions

This study presents the development of a composite gel, consisting of a biodegradable polymer matrix based on prolamins with increased incorporation of a silica precursor. The functional oxygen barrier of the gel in food packaging materials was effectively demonstrated when subjected to low relative humidity conditions, as evidenced by the oxygen transmission rate measurements. However, when precursor amounts were increased to 50 wt%, a slight decrease in the gel’s oxygen permeability was observed under higher relative humidity conditions. This can be attributed to the inherent property of silica particles to absorb moisture and subsequently safeguard the integrity of the gel matrix. The addition of precursor amounts did not enhance the gel’s barrier properties in low humidity environments, nor did it diminish the gel’s oxygen barrier properties in such conditions. Significantly, the incorporation of the silane precursor greatly improved water stability. The resulting silica particles interacted with hydroxyl groups in polysaccharides, polypeptides and citric acid reducing their interactions with water and restricting polymer chain movement. It was observed that higher precursor amounts improved mechanical resistance but also increased brittleness, as molecular movement of the polymer chains became hindered, and entanglements increased. This was accompanied by an increase in surface roughness due to particle agglomeration, which further supported the entanglement of the gels. Future research endeavors should prioritize the optimization of water stability and hygroscopic properties to achieve a reduced oxygen transmission rate, particularly in elevated humidity environments.

The cell compatibility of the gels was assessed through cytotoxicity testing using human keratinocytes and fibroblasts, according to DIN EN ISO 10993-5. Encouragingly, no cytotoxic effects were detected, indicating the safety of the gels for consumer contact. Furthermore, a leukocyte stimulation test was performed to evaluate the gel’s potential for causing inflammatory effects in end consumer contact scenarios. From the results obtained, it can be concluded that the gels are safe, based on the parameters of the selected bioassays. However, it is necessary to recognize that the leukocyte testing alone might not be sufficient to comprehensively evaluate the interaction of the gels with human skin. To ensure more comprehensive testing, for further research additional in vitro assays for possible skin sensitization and evaluation of allergenic potential is recommended. This step is crucial in assessing any potential risks related to sensitization and ensuring the overall safety and efficacy of the gels for consumer use.

Overall, this research contributes valuable insight to the development of composite gels for potential food packaging applications, demonstrating promising oxygen barrier properties and cytocompatibility. The study emphasizes the significance of thorough testing to guarantee the safety and suitability of these materials for human contact.

## 4. Materials and Methods

### 4.1. Materials

Wheat Gluten (CAS No. 8002-80-0) for prolamin extraction was purchased from Carl Roth (Karlsruhe, Germany) with a >75% protein content purity, d-Mannose > 99% (CAS No. 3458-28-4) was bought from Thermo Fisher Scientific (Waltham, MA, USA). The precursor tetraethyl orthosilicate (CAS No.78-10-4) and tetramethyl orthosilicate (CAS No. 681-84-5) were purchased with 98% purity from Sigma Aldrich (St. Louis, MO, USA). The citric acid (99.9%, CAS No. 77-92-9) was acquired from VWR International (Dresden, Germany). For the cell medium, the Dulbecco’s Modified Eagle Medium DMEM was bought from BioConcept Ltd. (Allschwil, Switzerland). The antibiotic-antimycotic solution (10,000 U/mL penicillin, 10,000 µm/mL streptomycin, 25 µg/mL amphotericin) was bought from PromoCell (Heidelberg, Germany), the fetal calf serum from PAN Biotech (Aidenbach, Germany) and the insulin (5 mg/mL) from PELOBiotech (Planegg, Germany). The Recombinant Human Fibroblast Growth Factor-2 (rhFGF2) was bought from Sartorius CellGenix GmbH (Freiburg, Germany), trypsin ethylenediaminetetraacetic acid and gentamycin (10 mg/mL) from Gibco, Life Technologies Limited (Thermo Fisher Scientific, Waltham, MA, USA). The bioassay ATPLite M Assay was purchased from PerkinElmer (Waltham, MA, USA) the cytotoxicity detection kit (LDH) from Roche (Basel, Switzerland) and the CAST^®^ ELISA for the sulfidoleukotrienes detection from Bühlmann Laboratories (Schönenbuch, Switzerland). The dextran solution was also obtained from Bühlmann Laboratories (Schönenbuch, Switzerland). For the preparation of the artificial sweat solution the natriumchlorid NaCl (>99.5%, CAS No. 7647-14-5), the hydrochloric acid 37% HCL (CAS No. 7647-01-0) and the sodium hydroxide NaOH (>99%, CAS No. 1310-73-2) were all purchased from Carl Roth (Karlsruhe, Germany) and the L-Histidine C_6_H_9_O_2_N_3 ∙_H_2_O (>98.5%, CAS No. 5934-29-2) from AppliChem GmbH (Darmstadt, Germany). The sodium dihydrogen phosphate dehydrate NaH_2_PO_4_ ∙ 2H_2_O (> 98%, CAS No. 13472-35-0) and artificial saliva for pharmaceuticals acquired from Sigma Aldrich (St. Louis, MO, USA). For extract sterile filtration a 0.2 µm filter from Sarstedt AG & Co.KG (Nümbrecht, Germany) and for blood storage EDTA monovettes were used (Sarstedt AG & Co.KG).

### 4.2. Preparation of Composite Gel

In a beaker, 20 g of wheat gluten were extracted with 200 mL of 70% ethanol. After stirring for 1 h the mixture was centrifuged with 4000 rpm for 10 min. The supernatant was used for further synthesis. 1 g of d-mannose was solved in 10 mL supernatant of the prolamin extraction. The precursors tetraethoxysilane and tetramethoxysilane were mixed in the volume ratio 30:70% *v*/*v*. Then 0.14 mL, 0.28 mL and 0.68 mL of this precursor was pipetted in the pre solution of prolamin and d-mannose. Anhydrous citric acid was added; the solution was stirred for 1 h and altered for 12 h at room temperature.

For the texture analysis, the sol-gels (ProMa) were weighed in at 6 g in a 70 × 40 mm Teflon cast and dried for 24 h in a desiccator filled with dry silica. For the water stability testing a volume of 1 mL of the solution was carefully pipetted into 25 mm^2^ Teflon casts and allowed to dry for a duration of 24 h.

### 4.3. IR Spectroscopy Studies

The chemical structure and composition of the gels were studied using the IR MB 3000, ABB Automation products GmbH (Alzenau, Germany). The gels were applied on double polishes silicon wafers, and dried at 80 °C in the oven for 30 min. The prepared samples were then analyzed with FT-IR spectroscopy, absorbance spectrum 4000–500 cm^−1^.

### 4.4. Tensile Strength Examinations of Composite Gels

The tensile strength of the gels was determined using the Texture Analyzer TA. XT2i, Stable Micro Systems Ltd. The films were punched with a stamping press into samples of 35 mm length, 2 mm width. For the sample with 50 wt% precursor it was not possible to solvent cast a stable film, the thickness was too high and the material to brittle. The samples were clamped into two brackets of the texture analyzer with gap distance of 20 mm. The thickness of each sample was measured before the experiment using a caliper gauge and multiplied with the height of the experiment design to determine the cross-section area of the samples. The samples were pulled apart with a speed of 1 mm/s until they break. The tensile strength stress σ and strain ε can be derived, this was determined from measured load and deflection the material sample, cross sectional area S0 and length l0. The stress strain curve was determined following Equations (1) and (2) [41].

(1) $$\sigma =\frac{F}{S_{0}}$$

(2) $$\varepsilon =\frac{dl}{l0}$$

### 4.5. Atomic Force Microscopy Analysis

The surface analysis of the gels was obtained with the MFP 3D-Classic, Asylum Research. The gels ProMa 0 wt% precursor and ProMa 50 wt% precursor were diluted 1:1 V% with 70% ethanol and applied in a thin layer on a polished silicon wafer, dried in an oven at 80 °C for 30 min. After drying observations were carried out using an MFP 3D-Classic Atomic Force Microscope (Asylum Research, Santa Barbara, CA, USA), operating in air under ambient humidity and room temperature conditions. Gels samples were examined in tapping mode with a resolution of 256 × 256. The drive frequency was 273.9 kHz. Areas of 1 × 1 micrometer and 10 × 10 micrometer were acquired, with an aluminum coated Micro Cantilever of 160 µm in length (160AC-NA, OPUS by MikroMasch, Sofia, Bulgaria) with a force constant 26 N/m (nominal value). The images were analyzed by MarSurf MfM Premium (Version 7.4.8737) according to ISO 25178 [96], including the calculation of roughness parameters.

### 4.6. Scanning Electron Microscopy Analysis

The morphology of the samples was analyzed on the surface and the cross section of dried gel samples. The gel samples were dispersed in 80%, 90%, 95% and 99% ethanol for each 5 min and dried using CPD 7501 Critical Point Drying Apparatus (Quorum Technologies, East Sussex, UK). The samples were gold sputtered with a turbomolecular-pumped sputter coater Quorum Q150V ES plus (Quorum Technologies, East Sussex, UK) and analyzed using Field Emission SEM Zeiss Gemini SEM 460 (Carl Zeiss AG, Oberkochen, Germany).

### 4.7. Rheological Investigations of Composite Gel

The viscosity of gel solutions was analyzed at different altering states with an Rheometer MCR 301 (Anton Paar GmbH, Graz, Austria) at T = +25 °C with a cone plate system (25 mm, 1 °C angle) following the measuring methodology of Mezger [97]. The measurement is shearing rate controlled with logarithmically increasing steps. With increasing shear rate, the measuring point duration was steadily shortened, starting at the shear rate of 0.001 Hz with 1000 s per measuring point and at the end of the experiment at the shear rate of 1000 Hz with only 1 s per measuring point. For the solutions altered for 1 d, 3 d, 7 d the measuring point duration was steadily shortened, starting at the shear rate of 0.001 1/s with 350 s per measuring point and at the end of the experiment at the shear rate of 1000 1/s with 10 s per measuring point.

### 4.8. Water Solubility of Composite Gels

The water solubility was determined in accordance to the protocol developed by Pearson [98]. After the drying process, the samples were weighed and subsequently immersed in 10 mL of deionized water for specified time intervals (1 min, 5 min, 15 min, 30 min, 60 min, 300 min). Following the immersion period, the water was filtered using a paper filter (Grade 392, Satorius AG, Göttingen, Germany), dried at room temperature for 24 h, and weighed again. The weight loss percentage was calculated by comparing the initial weight of the samples before immersion with the final weight after filtration and drying.

### 4.9. Gas Permeability of Composite Gels on PET

The gels were applied as a 4 µm wet film (dried film thickness 900 nm) onto a polyethylene foil (PET) with a thickness of 12 µm. Subsequently, the samples underwent a drying process at 80 °C for a duration of 30 min. After drying, the samples were carefully inserted into a Gas-Transmission Tester, manufactured by Brugger Feinmechanik GmbH. The testing procedure employed oxygen as the test gas following the ISO 15105-1 [99] guidelines and demonstrated from Startek et al. [64]. The chamber was maintained at a temperature of 23 °C, and the relative humidity was adjusted to 20%, 40%, 60%, and 80% relative humidity (r.H.).

### 4.10. In-Vitro Cytotoxicity Testing

Cell compatibility was assessed by preparing sample extracts following the DIN EN ISO 10993-12 [100] guidelines as previously reported [77]. To accomplish this, 200 mg samples (n = 3) of each material were mixed with either 10 mL of DMEM, 10 mL of a controlled sweat solution (5 g NaCl, 2.2 g NaH_2_PO_4_ ∙ 2H_2_O, 0.5g C_6_H_9_O_2_N_3_ ∙ H_2_O + HCL, 0.1 N NaOH—pH 5.5) or artificial saliva for pharmaceuticals (250 mL, Sigma-Aldrich) in Erlenmeyer flasks. The flasks were then shaken in a ThermoBath (GFL, Deutschland) for 24 h at 37 °C. Following the incubation period, any residual material was removed, and the extracts were sterilized using filtration through a 0.2 µm filter (Sarstedt AG & Co.KG).

Human keratinocytes HaCaT (provided by Prof. Fusenig, Heidelberg) were cultured in DMEM supplemented with 1% antibiotic-antimycotic solution and 10% fetal calf serum in 75 cm^2^ cell culture flasks. Normal human fibroblasts (PELOBiotech GmbH, Planegg, Germany) were cultured in DMEM supplemented with 500 µL insulin, 2.5 mL gentamycin, 250 µL rhFGF2 and 2% fetal calf serum. The cultures were maintained at 37 °C in a humidified atmosphere with 5% CO_2_ for seven days.

Afterwards, the cells were detached using trypsin ethylenediaminetetraacetic acid and seeded into 96-well plates at a cell density of 2 × 10^5^ cells/mL human keratinocytes and 1 × 10^5^ cells/mL fibroblasts. The plates were then incubated for 48 h. Subsequently, the medium was replaced with fresh medium, a controlled sweat solution or artificial saliva (negative control), material extracts, and Triton-X 100 (cytotoxicity control). The cells were incubated for 24 h.

Cell viability and proliferation were assessed using the ATPLite M Assay according to the manufacturer’s instructions. The cell count was calculated as a percentage of the negative control at each respective time point. The cytotoxic effects of the material were determined using the cytotoxicity detection kit according to the manufacturer’s instructions. Optical density was measured at 490 nm using a SPECTROstar-Omega plate reader (BGM Labtech). Cytotoxicity was calculated using Equation (3).

(3) $$\mathrm{cytotoxicity}\;[\%]=\frac{n-\mathrm{positive}\;\mathrm{control}}{\mathrm{negative}\;\mathrm{control}-\mathrm{positive}\;\mathrm{control}\cdot 100}$$

### 4.11. In-Vitro Inflammation Potential Testing

The inflammation potential was assessed using an enzyme immunoassay for the quantitative determination of sulfidoleukotrienes produced by isolated leukocytes.

Materials were dried and milled in fine particles and suspensions of 10 mg/mL, 0.1 mg/mL and 0.01 mg/mL were prepared in stimulation buffer. For particle control pure SiO_2_ particles were prepared following the same process as for the gels, a dispersion of 10 mg/mL was prepared. Particles were washed 5 times with distilled H_2_O to remove possible contaminants.

For the study, EDTA whole blood samples from two patients were collected [101] and stored in EDTA monovettes. The blood was transferred into 50 mL tubes, and 15 mL of the blood sample was mixed with 4 mL of dextran solution. The mixture was inverted and allowed to sediment for 30 min. After sedimentation, the supernatant was carefully collected using a Pasteur pipette and transferred into new 50 mL tubes. These tubes were then centrifuged at 160× *g* for 10 min to remove any remaining supernatant. The discarded supernatant was replaced with 15 mL of stimulation buffer, which was added to the isolated leukocytes. A volume of 200 µL of the leukocyte stimulation buffer solution was subsequently added to the samples and controls, with each determination performed in triplicate. The samples were sealed and incubated at 37 °C for 50 min. Following incubation, the samples were vortexed and centrifuged at 4 °C, 1000× *g* for 6 min. The supernatants from the triplicate determinations were combined (pooled). These pooled supernatants were then frozen and stored at −20 °C for further analysis.

The concentration of sulfidoleukotrienes (sLTC4, sLTD4, sLTE4) was determined using the CAST assay kit. The release of sulfidoleukotrienes (sLT) from the stimulation control and allergen was calculated using the following Equation (4):

(4) $$sLT=sLT\left[\alpha \right]\left(\frac{pg}{mL}\right)-sLT\left[backround\right](\frac{pg}{mL})$$

Here, “α” represents the control and samples.

The color absorbance was measured spectrophotometrically at a wavelength of 405 nm. For the quantification of sLTD4, a four-parameter fit regression model was used, with a calibration range of 50–3200 pg/mL. To determine a positive stimulation response, a threshold of ≥100 pg sLT/mL was considered after subtracting the value obtained from the negative control.

### 4.12. Statistical Analysis

IBM SPSS Software 29.0.0.0 was used to test the biological data for normal distribution, subsequently a Mann–Whitney-U-Test was carried out to determine the central tendencies of two independent random samples. Asterisks indicate significant deviations from the negative control (* *p* < 0.05; ** *p* < 0.01; *** *p* < 0.001).

## Figures and Tables

**Table 1:** Young modulus [MPa] of ProMa with 0/10/20 wt% precursor determines from stress strain curve.

	ProMa 0 wt% p.	ProMa 10 wt% p.	ProMa 20 wt% p.
Young Modulus MPa	10.01	17.12	21.07
SD	4.96	10.07	7.28

## Data Availability

Data available on request.
